# Oxidative stress drives liver failure during in vivo partial reprogramming

**DOI:** 10.1016/j.mocell.2026.100378

**Published:** 2026-06-04

**Authors:** Hee-Ji Eom, Beom-Ki Jo, Jumee Kim, Hyuk-Jin Cha

**Affiliations:** 1 College of Pharmacy, Seoul National University, Seoul, Republic of Korea; 2 College of Pharmacy, Sookmyung Women's University, Seoul, Republic of Korea; 3 Research Institute of Pharmaceutical Sciences, Seoul National University, Seoul, Republic of Korea

**Keywords:** In vivo reprogramming, Liver failure, N-acetylcysteine, Oxidative stress, Reactive oxygen species

## Abstract

In vivo reprogramming using the Yamanaka factors (OCT4, SOX2, KLF4, and c-MYC; OSKM) enables tissue regeneration but raises major safety concerns when factor expression is sustained. Here, using a doxycycline-inducible OSKM mouse model, we show that prolonged systemic OSKM induction causes early lethality associated with hepatocyte dedifferentiation and oxidative stress in the absence of tumor formation. Single-nucleus RNA sequencing revealed activation of reactive oxygen species (ROS), oxidative stress, and NRF2 signaling pathways in hepatocytes. Increased ROS production in hepatocytes, together with the higher resistance of female mice and sex-dependent differences in antioxidant response programs, implicates oxidative stress as a primary driver of mortality during sustained OSKM expression. Importantly, antioxidant treatment with N-acetylcysteine (NAC) alleviated oxidative stress and significantly improved survival without impairing reprogramming-associated cellular plasticity. These findings establish oxidative stress as a key driver of liver failure during sustained in vivo reprogramming and provide a mechanistic rationale for cyclic induction strategies.

## Graphical Abstract

**Figure:**
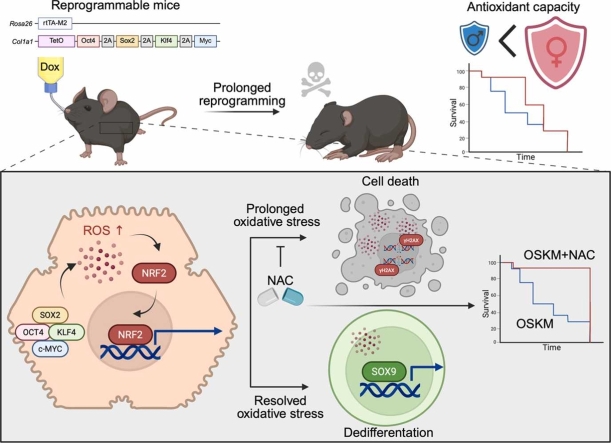

In vivo cellular reprogramming driven by the Yamanaka factors (OCT4, SOX2, KLF4, and c-MYC; OSKM) has emerged as a promising strategy for tissue regeneration and organismal rejuvenation (Jo et al., 2025b). Proof of concept for this approach has been demonstrated in multiple doxycycline-inducible OSKM mouse models (Jo et al., 2025b, Picó et al., 2025). However, sustained in vivo reprogramming is consistently associated with severe toxicity characterized by rapid weight loss and premature lethality (Ocampo et al., 2016), largely due to hepatic and intestinal failure (Parras et al., 2023). Similar systemic toxicity has also been reported in Caenorhabditis elegans following OSKM induction (Kamaludeen et al., 2024). Consequently, sustained systemic reprogramming—an essential requirement for whole-body rejuvenation—remains difficult to achieve safely. Current approaches therefore rely on cyclic OSKM induction or senescence-targeted reprogramming to reduce toxicity while preserving rejuvenation effects (Browder et al., 2022, Sahu et al., 2024).

As previously described (Jo and Song, 2025, Kim et al., 2023), systemic in vivo partial reprogramming was induced in OSKM-inducible mice (4Fk) by doxycycline administration (0.15 mg/mL in drinking water) (Figs. 1A and S1A). Consistent with previous findings (Parras et al., 2023), 4 days of continuous OSKM induction resulted in rapid weight loss and premature lethality (Fig. 1B), accompanied by marked elevation of plasma AST and ALT levels indicative of acute hepatocellular injury (Fig. 1C). To characterize early hepatic changes preceding death, livers were analyzed after 3 days of OSKM induction. *Sox9*, a marker of liver progenitor-like cells (LPLCs) (Li et al., 2023), was strongly induced, whereas hepatocyte markers including *Cyp2e1* and *Hnf4a* were markedly reduced, indicating hepatocyte dedifferentiation (Fig. 1D). Loss of HNF4α protein further supported this observation (Fig. S1B). In addition, markers of apoptosis (cleaved caspase-3) and DNA damage (γH2AX) were significantly increased in OSKM-induced livers (Figs. 1E, S1C, and S1D), consistent with severe hepatic injury during sustained reprogramming as extensively demonstrated (Parras et al., 2023). To explore molecular pathways associated with this phenotype, we analyzed previously generated single-nucleus RNA-sequencing data from OSKM-treated livers (GSE274988). In both acetaminophen (APAP) injury and OSKM induction, LPLCs, also described as transient regenerative progenitors (Jo et al., 2025b), were detected (Fig. 1F). However, in contrast to APAP injury, LPLC populations arising during OSKM induction showed strong enrichment of reactive oxygen species (ROS) and oxidative stress pathways, including activation of the KEAP1–NRF2 signaling axis, a central regulator of antioxidant responses (Fig. 1G, H, and Fig. S1E). Gene set enrichment analysis further confirmed enrichment of ROS- and NRF2-related signatures in OSKM-induced hepatocyte populations (Figs. 1I and S1F). Of note, the clear positive correlation between oxidative phosphorylation (OXPHOS) and oxidative stress module scores in LPLC1 and LPLC2 from OSKM-induced, but not APAP-induced, mice suggests that increased mitochondrial activity in OSKM-induced dedifferentiated hepatocytes may be accompanied by metabolic remodeling during liver regeneration (Caldez and Van Hul, 2018, Wang et al., 2024) and may contribute to oxidative stress (Fig. S1G). Despite extensive intestinal cell death, indicated by increased cleaved caspase-3 and phosphorylated p38 (Fig. S1H), single-cell RNA-sequencing analysis of intestinal tissue (GSE238057) revealed that newly generated revival stem cell-like cells (revSCs) and atrophy-induced villus epithelial cells (aVECs) did not display enrichment of oxidative stress or NRF2 gene signatures (Fig. S1I-J). Instead, oxidative stress signatures were primarily detected in mature enterocytes (Fig. S1K-M). These results suggest that sustained OSKM induction induces prominent oxidative stress signatures in hepatic LPLCs, while intestinal progenitor-like populations show limited enrichment.

**Fig. 1:**
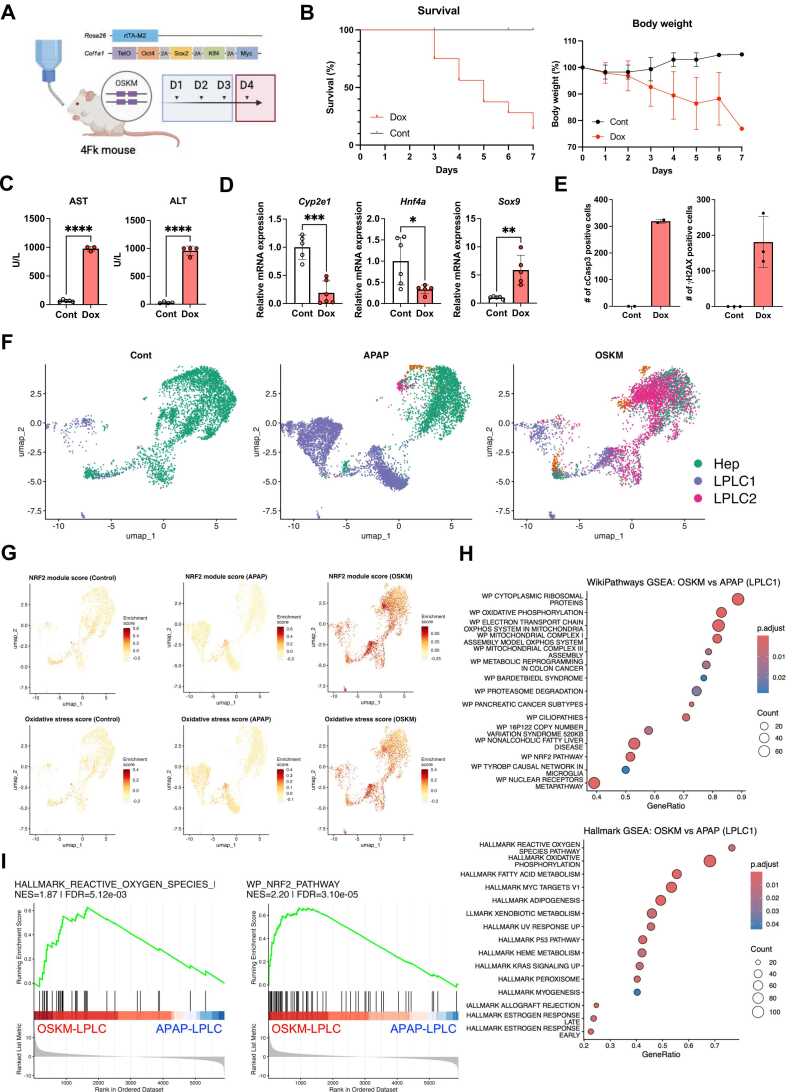
*Sustained OSKM expression drives hepatic failure through liver-specific induction of oxidative stress.* (A) Schematic illustration of doxycycline (Dox)-inducible OSKM expression in homozygous 4Fk mice and experimental timeline for continuous OSKM induction. (B) Kaplan-Meier survival curve (left) (Cont *n* = 8, Dox *n* = 16) and body weight changes (right) (Cont *n* = 6, Dox *n* = 14) of control and OSKM-induced mice during continuous OSKM induction. (C) Plasma levels of aspartate aminotransferase (AST) and alanine aminotransferase (ALT) in control and OSKM-induced mice. (D) Relative mRNA expression of hepatocyte marker genes (*Cyp2e1* and *Hnf4α*) and LPLC marker gene (*Sox9*) in livers following continuous OSKM induction. All data were normalized to 18S rRNA as the internal reference, and relative expression values were calculated using the comparative ΔΔCt method relative to the indicated control group. (E) Quantification of cleaved caspase-3 and γH2AX-positive nuclei in liver sections following OSKM induction. Positive nuclei were counted per 10x objective field (6 fields per section, 2 ∼ 3 mice per group). (F) UMAP visualization of hepatocytes and liver progenitor-like cell populations (LPLC1 and LPLC2) in control, acetaminophen (APAP)-treated, and OSKM-induced livers based on single-nucleus RNA sequencing. (G) Feature plots showing NRF2 module scores and oxidative stress scores across hepatocyte populations in control, APAP-treated, and OSKM-induced livers. (H) Gene set enrichment analysis (GSEA) showing enrichment of oxidative stress– and NRF2-related pathways in LPLC1 cells from OSKM-induced livers compared with APAP-induced injury. (I) GSEA plots validating enrichment of reactive oxygen species (ROS) and NRF2 signaling pathways in OSKM-induced hepatocytes.

To validate oxidative stress responses in hepatocytes, whole livers and primary hepatocytes were isolated after 3 days of OSKM induction (Fig. 2A). Activation of the NRF2 pathway was confirmed by increased expression of canonical target genes, including *Nqo1, Gsr,* and *Srxn1,* in OSKM-expressing livers, compared with rtTA controls (Fig. 2B). Compared with hepatocytes from rtTA-only control mice, hepatocytes from doxycycline-treated 4Fk mice exhibited increased apoptosis, elevated DNA damage (γH2AX), and activation of stress signaling as indicated by phosphorylation of p38 (Fig. 2C). Isolated hepatocytes also showed induction of NRF2-responsive genes and the ROS-responsive gene *Chchd2* (Liu et al., 2024) (Fig. 2D), accompanied by increased NRF2 protein levels (Fig. 2E). Time-course analysis demonstrated progressive NRF2 accumulation and upregulation of downstream target genes during sustained OSKM induction (Fig. 2F and G), indicating that oxidative stress arises cell-autonomously within reprogramming hepatocytes. Because ROS production is an intrinsic feature of early cellular reprogramming (Zhou et al., 2016), we hypothesized that hepatocyte-derived ROS contributes directly to liver injury during sustained in vivo reprogramming. Intracellular ROS levels were therefore measured in isolated hepatocytes using DCF-DA. Flow cytometry revealed a reduction in the viable cell population together with a marked increase in ROS-positive hepatocytes following OSKM induction (Fig. 2H), indicating elevated ROS production concurrent with hepatocyte death.

**Fig. 2:**
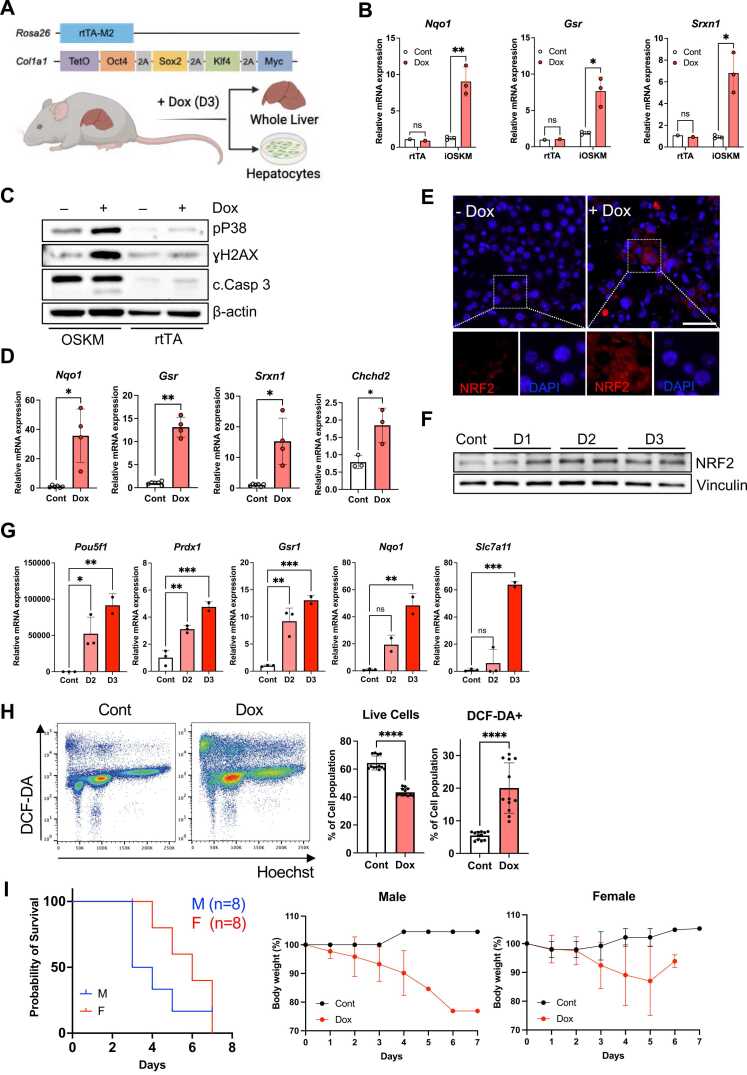

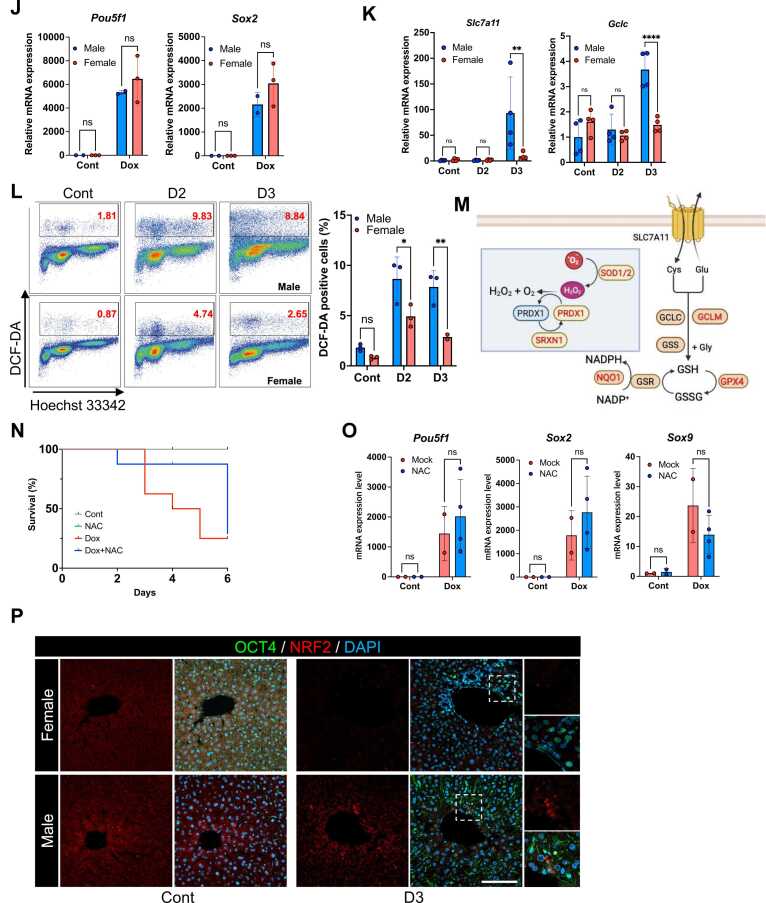
*Antioxidant intervention rescues OSKM-induced hepatic failure by mitigating cell-autonomous oxidative stress in hepatocytes.* (A) Experimental schematic for isolation of whole liver lysates and primary hepatocytes after 3 days of continuous OSKM induction. (B) Relative mRNA expression of canonical NRF2 downstream genes (*Nqo1*, *Gsr*, and *Srxn1*) in whole livers from control and OSKM-induced mice. All data were normalized to 18S rRNA as the internal reference, and relative expression values were calculated using the comparative ΔΔCt method relative to the indicated control group. (C) Immunoblot analysis of p-p38 MAPK, p-H2A.X and cCasp3 in primary hepatocytes isolated from rtTA^Tg/Tg^ and iOSKM mice with or without Dox administration. (D) Relative mRNA expression of NRF2 target genes (*Nqo1*, *Gsr*, *Srxn1*) and the ROS-responsive gene *Chchd2* in isolated hepatocytes following OSKM induction. All data were normalized to 18S rRNA as the internal reference, and relative expression values were calculated using the comparative ΔΔCt method relative to the indicated control group. (E) Immunofluorescence staining of NRF2 (red) in hepatocytes from control and OSKM-induced mice. DAPI (blue) marks nuclei. Scale bar = 50 μm. (F) Immunoblot analysis of NRF2 protein levels in livers during time-dependent continuous OSKM induction. (G) Time-dependent mRNA expression of *Pou5f1* and NRF2 downstream genes (*Prdx1, Gsr1, Nqo1*, and *Slc7a11*) Data represent the mean with SD (*n* = 3). (D2 = 48 h after doxycycline administration; D3 = 72 h after doxycycline administration) All data were normalized to 18S rRNA as the internal reference, and relative expression values were calculated using the comparative ΔΔCt method relative to the indicated control group. (H) Flow cytometric analysis of intracellular ROS levels in isolated hepatocytes using DCF-DA staining. Representative pseudocolor plots (left) and quantification of live cells (Hoechst-positive) and DCF-DA–positive hepatocytes (right) following OSKM induction. Data represent the mean with SD (*n* = 12). (I) Body weight changes (left) (Male Cont *n* = 1, Male Dox *n* = 6, Female Cont *n* = 5, Female Dox *n* = 8) and Kaplan-Meier survival curves (right) (Male *n* = 8, Female *n* = 8) of age-matched male (M) and female (F) mice subjected to continuous OSKM induction. (J) Relative mRNA expression of *Pou5f1* and *Sox2* in livers from male and female mice following OSKM induction. All data were normalized to 18S rRNA as the internal reference, and relative expression values were calculated using the comparative ΔΔCt method relative to the indicated control group. (K) Relative mRNA expression of select NRF2 downstream genes (*Slc7a11* and *Gclc*) in hepatocytes isolated from male and female mice during OSKM induction. (D2 = 48 h after doxycycline administration; D3 = 72 h after doxycycline administration) (*n* = 4). All data were normalized to 18S rRNA as the internal reference, and relative expression values were calculated using the comparative ΔΔCt method relative to the indicated control group. (L) Flow cytometric analysis of ROS production in hepatocytes from male and female mice using DCF-DA staining following continuous OSKM induction. Representative pseudocolor plots (left) and quantification of DCF-DA–positive hepatocytes (right). Data represent the mean with SD (*n* = 3). (D2 = 48 h after doxycycline administration; D3 = 72 h after doxycycline administration) (M) Schematic illustration of major ROS detoxification pathways and glutathione-dependent antioxidant systems in hepatocytes. Genes shown in red indicate significantly higher basal mRNA expression levels in female hepatocytes compared with male hepatocytes. (N) Kaplan-Meier survival analysis of mice subjected to continuous OSKM induction with or without N-acetylcysteine (NAC) treatment (*N* = 22). (O) Relative mRNA expression of reprogramming-associated genes (*Pou5f1*, *Sox2*, and *Sox9*) in livers from OSKM-induced mice treated with or without NAC. All data were normalized to 18S rRNA as the internal reference, and relative expression values were calculated using the comparative ΔΔCt method relative to the indicated control group. (P) Immunofluorescence staining of NRF2 (red) and OCT4 (green) in liver sections of male and female 4Fk mice with or without continuous OSKM induction. Dotted lines indicate OCT4-positive cells. DAPI stains the nuclei. Scale bar = 100 μm.

Interestingly, female mice exhibited significantly greater resistance to premature lethality than age-matched male mice following continuous OSKM induction (Fig. 2I), despite similar levels of OSKM expression and hepatocyte dedifferentiation (Figs. 2J and S2A). Female hepatocytes showed reduced induction of selected NRF2 downstream genes, including *Slc7a11* and *Gclc,* while other NRF2 targets were similarly induced in both sexes (Figs. 2K and S2B). Direct measurement of ROS further revealed significantly fewer ROS-positive hepatocytes in female mice (Fig. 2L). In addition, basal expression of antioxidant genes involved in ROS detoxification and glutathione metabolism (Fig. 2M) was higher in female hepatocytes (Fig. S2C-E). These findings suggest that reduced ROS accumulation contributes to the increased resistance of female mice to OSKM-induced liver injury.

Given the prominent oxidative stress observed in hepatocytes, we next tested whether antioxidant treatment could mitigate OSKM-induced toxicity. Administration of N-acetylcysteine (NAC), a clinically used antioxidant, significantly delayed the onset of premature lethality during continuous OSKM induction (Fig. 2N). Importantly, NAC treatment did not impair OSKM expression or hepatocyte dedifferentiation, as assessed by *Pou5f1, Sox2,* and *Sox9* expression (Fig. 2O). NAC also reduced hepatocyte DNA damage (γH2AX) and attenuated NRF2 nuclear accumulation during OSKM induction (Fig. S2F and G). These findings suggest that high ROS production is dispensable for transient hepatocyte reprogramming. Moreover, the loss of HNF4α associated with OCT4 induction during Dox treatment was restored within 24 h after Dox withdrawal, concomitant with a marked reduction in OCT4-positive cells (Fig. S2E), indicating that OSKM-induced hepatocyte dedifferentiation is transient and rapidly reversible. Consistent with our transcriptomic findings, NRF2 nuclear localization was prominent in male livers but minimal in female livers, whereas intestinal tissues showed little NRF2 induction or activation under identical OSKM induction conditions (Figs. 2P, S2I, and J).

Together, these findings identify excessive oxidative stress as a primary driver of hepatic failure during sustained in vivo OSKM reprogramming. While transient ROS signaling is compatible with early metabolic remodeling, prolonged OSKM expression overwhelms hepatocyte redox homeostasis, leading to DNA damage, apoptosis, and acute liver failure. Partial rescue by NAC further supports a causal role for ROS in reprogramming-induced toxicity. Notably, Sox9, Pou5f1, and Sox2 expression levels were largely comparable with NAC treatment (Fig. 2O), suggesting limited effects on OSKM-induced reprogramming marker expression. The enrichment of OXPHOS signatures in OSKM-induced LPLCs (Fig. S1G), together with previous studies linking mitochondrial oxidative metabolism to liver regeneration (Caldez and Van Hul, 2018, Wang et al., 2024), raises the possibility that increased mitochondrial activity may participate in hepatic reprogramming. Previous studies have shown that cyclic OSKM induction (2 days on and 5 days off) can mitigate premature lethality while preserving rejuvenation effects, including reversal of aging-associated epigenetic changes (Browder et al., 2022). Consistently, NRF2 downstream genes (Fig. 2K) were induced together with hepatic reprogramming marker genes at day 3 (Fig. S2A), suggesting a temporal association between NRF2 activation and hepatic reprogramming. However, direct experimental comparison between cyclic and sustained in vivo reprogramming will be required to validate this model. Because hepatocytes exhibit particularly high reprogramming competence (Jo and Song, 2025, Jo et al., 2025b), other tissues subjected to prolonged OSKM induction may similarly experience stress-related toxicity once their tolerance thresholds are exceeded.

Collectively, these findings indicate that optimization of OSKM induction schedules should consider the balance between regenerative dedifferentiation and oxidative stress responses. Antioxidant intervention such as N-acetylcysteine may therefore represent a practical strategy to reduce redox-driven toxicity during in vivo reprogramming. Careful tuning of reprogramming duration will be essential to harness regenerative benefits while minimizing systemic risk during rejuvenation strategies.

## CRediT authorship contribution statement

**Hee-Ji Eom:** Writing – review & editing, Writing – original draft, Investigation. **Beom-Ki Jo:** Writing – review & editing, Writing – original draft, Visualization, Investigation. **Jumee Kim:** Validation, Investigation. **Hyuk-Jin Cha:** Writing – review & editing, Writing – original draft, Supervision, Funding acquisition, Conceptualization.

## Declaration of Competing Interests

The authors declare that they have no known competing financial interests or personal relationships that could have appeared to influence the work reported in this paper.

## Footnotes

Appendix A Supplementary data associated with this article can be found in the online version at doi:10.1016/j.mocell.2026.100378.
