# Supplementary material for: Oxidative stress drives liver failure during in vivo partial reprogramming
Source: Mol Cells. 2026 Jun 4;49(8):100378. doi: 10.1016/j.mocell.2026.100378 (PMC13330666; doi:10.1016/j.mocell.2026.100378)
Supplement: Supplementary file 1 — Supplementary material [file mmc1.docx]

**Oxidative Stress Drives Liver Failure During In Vivo Partial Reprogramming**
Hee-Ji Eom¹*, Beom-Ki Jo¹*, Jumee Kim², and Hyuk-Jin Cha¹,³#
¹ College of Pharmacy, Seoul National University, Seoul, Republic of Korea
² College of Pharmacy, Sookmyung Women’s University, Seoul, Republic of Korea
³ Research Institute of Pharmaceutical Sciences, Seoul National University, Seoul, Republic of Korea

* These authors contributed equally.
# Correspondence: Hyuk-Jin Cha (hjcha93@snu.ac.kr)

## **Contents of this Supplementary Document**

## This supplementary document includes:

## Materials and Methods

## Supplementary Figures (Figures S1–S2)

## Supplementary Figure Legends

**Materials and Methods**

**Mice**

Col1A1-TetO-OSKM;ROSA26-rtTA mice (4Fk mice) were obtained from the Jackson Laboratory (no.011004). ROSA26rtTA mice were generated from Col1A1TetO-OSKM;ROSA26rtTA mice. Mice used for all experiments were 8 - 12 weeks of age and housed in conventional cage with 12h - 12h light–dark cycle and access to food and water *ad libitum*, under pathogen-free conditions at the Seoul National University. Both male and female mice were included in the study, but a single sex was used within each experimental batch to control for sex-related variability. For continuous OSKM expression, doxycycline hyclate (Sigma, D9891) was administered in drinking water at 0.15 mg/ml with 5% sucrose (Samchun Chemicals, 50010). The solution was freshly prepared and replaced at least every 3 days. For NAC treatment, N-Acetyl-L-cysteine (Sigma, A9165) was administered daily via intraperitoneal (IP) injection at a dose of 200 mg/kg, prepared in DPBS with pH adjusted using NaOH.

**Hepatocyte isolation**

Mice were anaesthetized with Avertin (250 mg/kg, IP injection) before surgical procedure. After anaesthetization, hepatocyte isolation proceeded with well-established two-step collagenase perfusion technique with some adjustments. Concisely, 24-gauge syringe was inserted to a tube connected to the peristaltic pump and cannulated the portal vein. After cannulation, vena cava was cut for allowing blood flow. 10 ml of EDTA buffer was perfused to wash out the blood from liver and 20 ml of collagenase buffer (1 mg/ml) was perfused for digestion. During perfusion, several times of clamping the vena cava with forceps for 5 - 10 s were conducted for efficient perfusion to all liver vasculatures. The liver was dissected and moved to Petri dish with 20 ml of hepatocyte culture media and ruptured with forceps to release hepatic cells. All hepatic cells were filtered through 70 µm cell strainer. Cells were centrifuged at 50 g for 2 min at 4 °C and the supernatant were discarded to eliminate non-parenchymal cells. Hepatocyte-enriched pellet was resuspended in HBSS and centrifuged at 50 g for 2 min at 4 °C for washing. To enrich the live hepatocyte, 9 ml of Percoll (Sigma, P1644) mixed with 2 ml of 10X PBS was added to 9 ml of HBSS containing hepatocytes. Cells were centrifuged at 200 g for 5 min at 4 °C. Live hepatocytes were kept for further analysis or culture.

**In vitro culture of primary hepatocytes**

For in vitro culture of primary hepatocytes, isolated primary hepatocytes were counted using hemacytometer after trypan blue staining and 1×10⁶ cells were seeded in collagen-coated 60 pi culture plates. For maintenance of primary hepatocytes, cells were cultured in Dulbecco’s modified Eagle’s medium (DMEM) supplemented with 10% FBS and 1% penicillin/streptomycin. Culture media was changed 3 hours after seeding and changed every day. For OSKM induction, 1 µg/ml of doxycycline was treated in the culture media.

**Immunofluorescence**

Liver tissues were fixed in 4% paraformaldehyde (PFA) at 4 °C overnight, followed by dehydration in 30% sucrose at 4 °C overnight. Samples were then embedded in Tissue-Tek optimal cutting temperature (OCT) compound (Sakura Finetek) and frozen for cryosectioning. For immunofluorescence, 6 µm frozen sections were treated with PBST (0.1% Triton X-100 in DPBS) to remove remaining OCT compound. For antigen retrieval, slides were kept in tris-EDTA buffer (pH 9.0) at 95 °C for 20 min and cooled down for 45 min. Retrieved sections were quickly washed with distilled water and blocked with 4% bovine serum albumin (BSA) in PBST for 1 hour. Then, slides were incubated with primary antibody at 4 °C overnight and treated with secondary antibodies at room temperature for 2 hours. 4′,6-Diamidino-2-phenylindole (DAPI) staining was conducted for nuclear counterstaining. Slides were covered with slide glass using MOWIOL mounting solution. Fluorescence images were acquired using either a widefield fluorescence microscope (Olympus BX53) or a confocal laser scanning microscope (Leica TCS SP8). Following antibodies were used for immunostaining: HNF4a (Abcam, ab41898, 1:400), Cleaved Caspase-3 (Cell Signaling Technology, #9661, 1:400), p-H2A.X (Cell Signaling Technology, #9718, 1:400), NRF2 (Cell Signaling Technology, #12721, 1:500), OCT4 (BD, 611203, 1:500) and Lamin A/C (Santa Cruz Biotechnology, sc-376248, 1:400).

**Plasma analysis**

Mouse blood was collected from the submandibular vein and left at room temperature for at least 30 min to clot. Then the samples were centrifuged at 2000 g for 10 min at 4 °C and the supernatant (plasma) were obtained for further analysis. Biochemical analyses for alanine aminotransferase (ALT) and aspartate aminotransferase (AST) were conducted as indicators of liver function using the DRI-CHEM 4000i (Fuji Film, Tokyo, Japan).

**RNA Extraction and RT-qPCR**

Total RNA was extracted from mouse liver tissues and primary hepatocytes using the easy-BLUE Total RNA Extraction Kit (iNtRON Biotechnology, 17061). Complementary DNA (cDNA) was synthesized from total RNA samples using the PrimeScript RT Reagent Kit (TAKARA, RR036A) according to the manufacturer’s insturctions. RT-qPCR was performed on a QuantStudio 3 Real-Time PCR System (ThermoFisher Scientific) using TB-green Premix Ex Taq (TAKARA, RR420), following the manufacturer’s protocol. All data were normalized to 18S rRNA as the internal reference, and relative expression values were calculated using the comparative ΔΔCt method relative to the indicated control group.

**Immunoblotting**

Liver tissues or hepatocytes were lysed in ice-cold radioimmunoprecipitation assay (RIPA) buffer containing 1% protease inhibitor cocktail and 0.1% sodium orthovanadate for 1 hour with periodic vertexing. Lysates were centrifuged at 14,000g for 20 min at 4 °C. With the supernatants, total protein concentration was analyzed using Pierce™ BCA protein assay kit (ThermoFisher Scientific, #23225). 10 to 20 µg of total protein was electrophoresed on sodium dodecyl sulfate-polyacrylamide gels of various acrylamide concentrations (7.5%, 10%, and 12%). Separated proteins were transferred to polyvinylidene difluoride (PVDF) membranes (Sigma, #IPVH00010). The membranes were blocked with 5% Difco™ Skim Milk (BD, #232100) in TBST (Tris-buffered saline with 0.1% Tween-20) for 1 hour at room temperature and washed three times with TBST for 10 min each. Membranes were incubated overnight at 4 °C with the primary antibody (1:1000) in TBST supplemented with 0.1% sodium azide. After incubation, membranes were washed three times with TBST for 10 min each and incubated with HRP-conjugated secondary antibody (Jackson Immunoresearch Laboratories) in TBST for 1 hour at room temperature. Membranes were washed three times with TBST for 15 min each and developed using the WEST-Queen kit (iNtRON Biotechnology, #16026) on a ChemiDoc system. Antibodies used were Cleaved Caspase-3 (Cell Signaling Technology, #9661), p-H2A.X (Cell Signaling Technology), p-p38MAPK (Cell Signaling Technology, #9718), NRF2 (Cell Signaling Technology, #12721), beta-actin (Santa Cruz Biotechnology, sc-47778), and Vinculin (Santa Cruz Biotechnology, sc-25336).

**Flow cytometry**

Isolated primary hepatocytes were washed with DPBS. stained with 10 µM H2DCFDA (Sigma, #287810) and 15 µg/mL Hoechst 33342 were added to the samples and incubated for 30 min at 37 °C with periodic voltexing every 10 minutes. Stained hepatocytes were washed with DPBS three times and analyzed with FACSCelesta (BD Biosciences). The data were analyzed with FlowJo™ software (BD Biosciences).

## **snRNA-seq Data Processing and UMAP Visualization**

## Previously generated single-nucleus RNA-sequencing (snRNA-seq) data (GSE274988) {Jo, 2025 #320} were reanalyzed using R (version 4.5.1) in RStudio. Processed count matrices and accompanying metadata were imported into Seurat (version 5.4.0) for downstream analysis. Cells with low gene counts or high mitochondrial gene expression were excluded based on standard quality control criteria.

## Data were log-normalized and scaled using the Seurat workflow. Highly variable genes were identified using the FindVariableFeatures function, followed by principal component analysis (PCA). The top principal components were used to construct a shared nearest neighbor graph, and Uniform Manifold Approximation and Projection (UMAP) was performed using the RunUMAP function for visualization. Cluster identities were assigned based on previously annotated cell types and canonical marker gene expression.

## **Gene Set Enrichment Analysis**

## Differentially expressed genes (DEGs) between experimental conditions were identified using the FindMarkers function in Seurat. Gene set enrichment analysis was performed in R using the clusterProfiler package (version 4.16.0) with gene sets obtained from the Molecular Signatures Database (MSigDB). Hallmark and WikiPathways gene sets were used for pathway enrichment analysis. Enrichment results were visualized using normalized enrichment scores (NES) and adjusted p-values (Benjamini–Hochberg correction).

**Statistical analysis**

All data are presented as mean ± standard error of the mean (SEM). Statistical analyses were performed using GraphPad Prism (version 10; GraphPad Software). Comparisons between two groups were conducted using unpaired two-tailed Student’s t-test. For comparisons involving more than two groups, one-way ANOVA followed by Tukey’s post hoc test was used. Significance was set as P < 0.05 (*), P < 0.01(**), P < 0.001 (***).

**Supplementary Figure legends**

**Figure S1. (A)** Relative mRNA expression of Pou5f1, Sox2, Klf4, and Myc in livers sampled from control and OSKM-induced mice. Data represent the mean with SD (n = 3). All data were normalized to 18S rRNA as the internal reference, and relative expression values were calculated using the comparative ΔΔCt method relative to the indicated control group. **(B)** Immunofluorescence staining of HNF4α (green) in liver sections from control (Cont) and doxycycline-induced (Dox) mice at day 3 of continuous OSKM induction. DAPI (blue) marks nuclei. Quantification of HNF4α-positive cells is shown on the right. Scale bar = 50 μm **(C-D)** Representative immunofluorescence images, showing hepatocyte cell death (cleaved Caspase 3: cCasp3) and stress marker (γH2AX) staining in control and OSKM-induced livers (left), and quantification of positive nuclei (right). Scale bar = 100 μm **(E)** Dot plot showing enrichment of Hallmark and WikiPathways gene sets in LPLC2 populations from OSKM-induced livers compared with acetaminophen (APAP)-induced injury. **(F)** Gene set enrichment analysis (GSEA) plots demonstrating enrichment of reactive oxygen species (ROS) and NRF2 signaling pathways in LPLC2 cells from OSKM-induced livers. **(G)** Scatter plots showing the relationship between OXPHOS module scores (x-axis) and oxidative stress module score (y-axis) at single cell resolution in LPLC1 (top) and LPLC2 (bottom) populations under APAP (green) and OSKM (red) conditions. Module scores were calculated using Seurat’s AddModuleScore based on the Hallmark gene sets. Dashed lines indicate linear regression fits with 95% confidence intervals. Spearman’s rank correlation coefficient (rho), p-value, and cell number (n) are shown for each panel **(H)** Immunoblot analysis of cleaved caspase-3 (cCasp3), phosphorylated p38 (pP38), and β-actin in liver lysates collected at the indicated time points during continuous OSKM induction. **(I)** UMAP visualization of intestinal cell populations in control and OSKM-induced conditions, including revival stem cell-like cells (revSCs) and atrophy-induced villus epithelial-like cells (aVECs). **(J)** Dot plots showing Hallmark and WikiPathways enrichment analyses for revSCs and aVECs following OSKM induction relative to control conditions. **(K)** Feature plots showing NRF2 module scores and oxidative stress scores across intestinal epithelial cell populations under control and OSKM-induced conditions. **(L-M)** Quantification of NRF2 and oxidative stress module scores in revSCs, aVECs, mature enterocytes (mECs), and early enterocytes (eECs), indicating no significant enrichment of oxidative stress– or NRF2-related signatures in intestinal cell populations.

**Figure S2. (A)** Relative mRNA expression of hepatocyte dedifferentiation markers Epcam and Cd44 in livers from male and female mice under control conditions and after 2 or 3 days of continuous OSKM induction, showing comparable induction between sexes. Data represent the mean with SD (n = 4). All data were normalized to 18S rRNA as the internal reference, and relative expression values were calculated using the comparative ΔΔCt method relative to the indicated control group. **(B)** Relative mRNA expression of NRF2 downstream antioxidant genes (Nqo1, Gsr, Prdx1, and Srxn1) in livers from male and female mice following OSKM induction. Data represent the mean with SD (n = 4). All data were normalized to 18S rRNA as the internal reference, and relative expression values were calculated using the comparative ΔΔCt method relative to the indicated control group. **(C)** Relative mRNA expression of NRF2 downstream antioxidant genes involved in glutathione-mediated detoxification (Sod1/2, Prdx1, and Srxn1) in livers from male and female mice following OSKM induction. Data represent the mean with SD (n = 6). All data were normalized to 18S rRNA as the internal reference, and relative expression values were calculated using the comparative ΔΔCt method relative to the indicated control group. **(D)** Relative mRNA expression of NRF2 downstream antioxidant genes involved in glutathione-mediated detoxification (Gclm, Gclc, Gsr, and Nqo1) in livers from male and female mice following OSKM induction. Data represent the mean with SD (n = 6). All data were normalized to 18S rRNA as the internal reference, and relative expression values were calculated using the comparative ΔΔCt method relative to the indicated control group. **(E)** Relative mRNA expression of NRF2 downstream antioxidant genes involved in lipid peroxide detoxification (Slc7a11, and Gpx4) in livers from male and female mice following OSKM induction. Data represent the mean with SD (n = 6). All data were normalized to 18S rRNA as the internal reference, and relative expression values were calculated using the comparative ΔΔCt method relative to the indicated control group. **(F)** Immunofluorescence staining of γH2AX (red), HNF4α (green) in liver sections of 4fk mice with or without continuous OSKM induction. DAPI stains the nuclei. **(G)** Immunofluorescence staining of NRF2 (red), LMNA (green) in liver sections of 4fk mice with or without continuous OSKM induction. DAPI stains the nuclei. **(H)** Immunofluorescence staining of HNF4α (red) and OCT4 (green) in liver sections sampled from control (Cont), doxycycline-treated (Dox), and doxycycline-withdrawn (Dox + Rest 24h) mice. DAPI stains the nuclei. Scale bar = 100 μm **(I)** Immunoblot analysis of NRF2, OCT4 and β-actin levels in isolated intestinal epithelial cell lysates from control (Cont) and doxycycline-treated (Dox) mice. **(J)** Immunofluorescence staining of NRF2 (red) and OCT4 (green) in liver sections of male and female 4fk mice with or without continuous OSKM induction. DAPI stains the nuclei. Scale bar = 100 μm
