# Supplementary figures and images for: Oxidative stress drives liver failure during in vivo partial reprogramming
Source: Mol Cells. 2026 Jun 4;49(8):100378. doi: 10.1016/j.mocell.2026.100378 (PMC13330666; doi:10.1016/j.mocell.2026.100378)

Figure S1

A

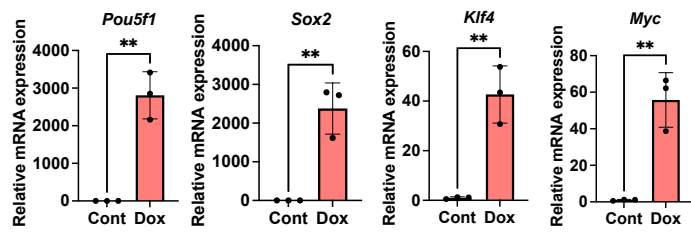

B

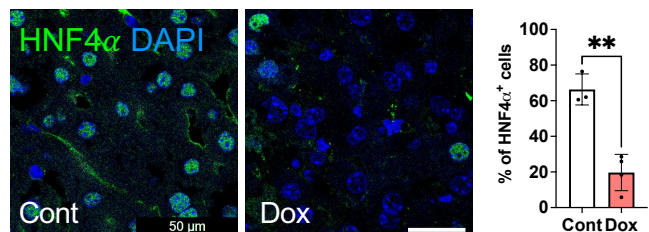

C

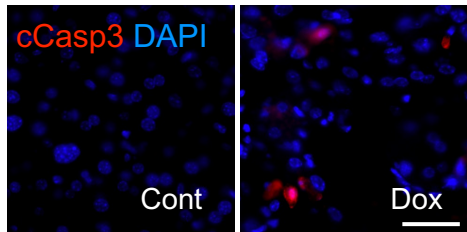

D

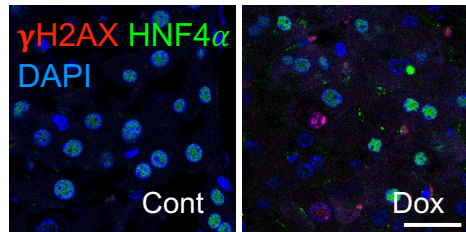

E

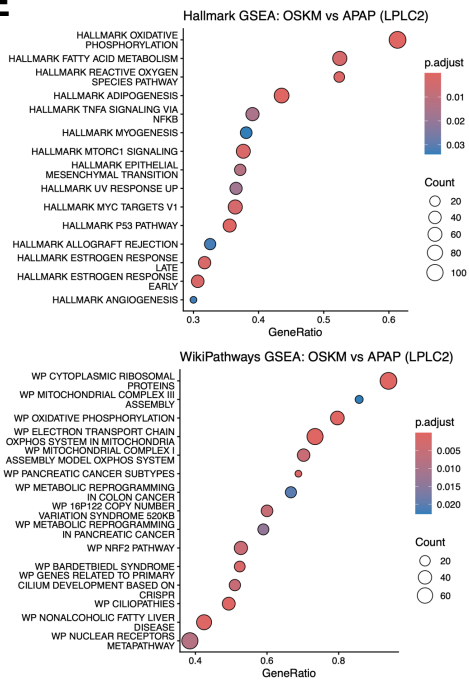

F

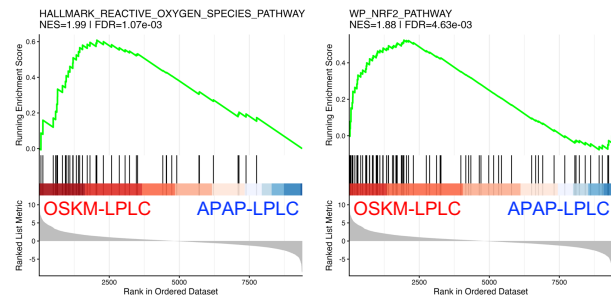

G

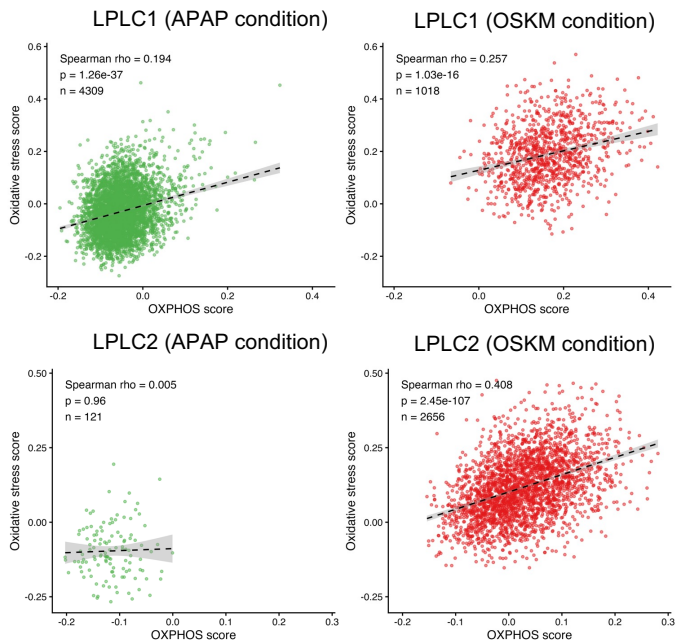

Figure S1

H

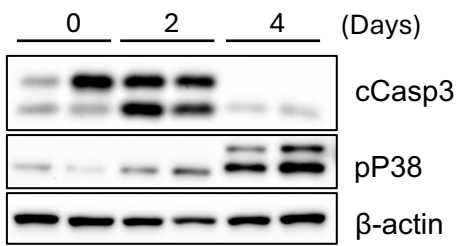

I

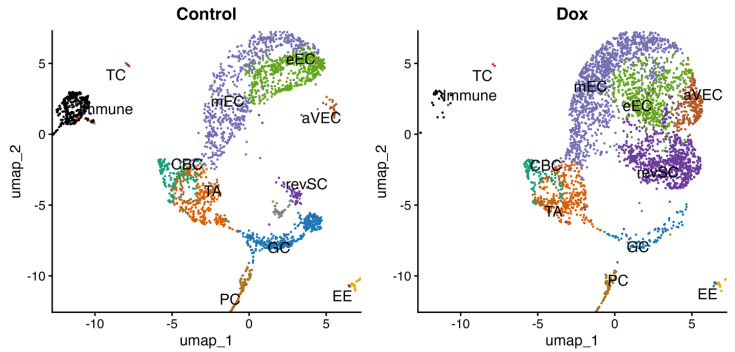

J

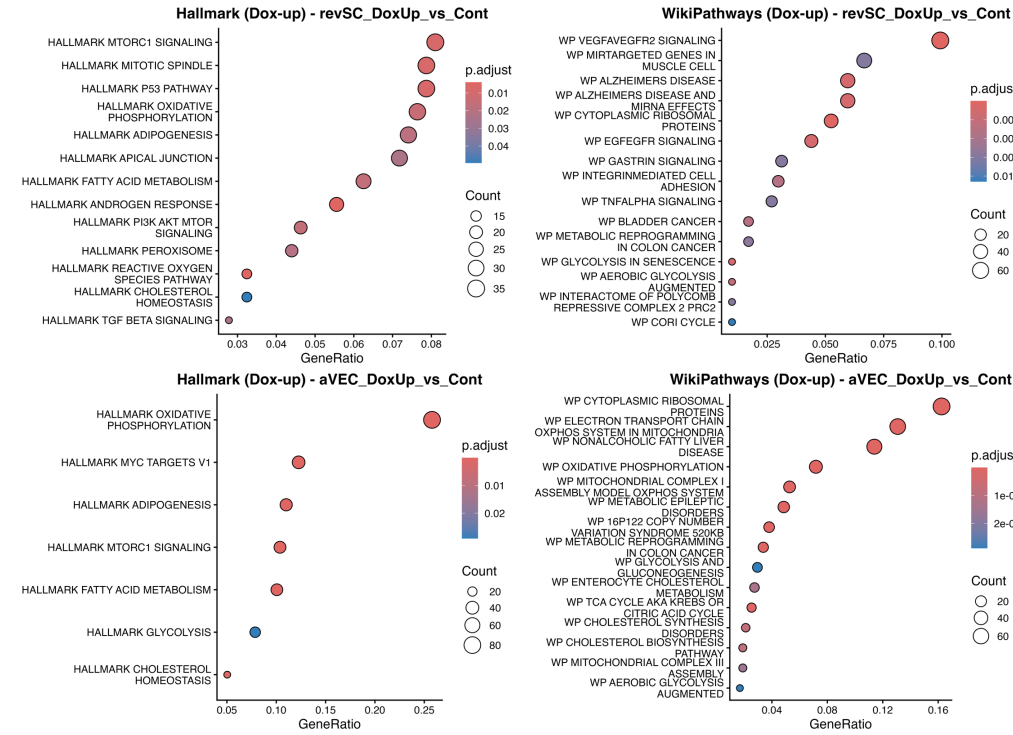

K

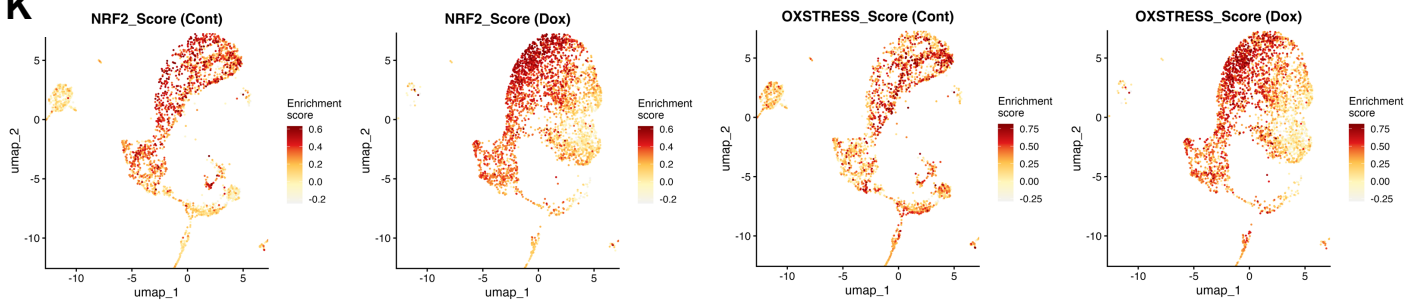

L

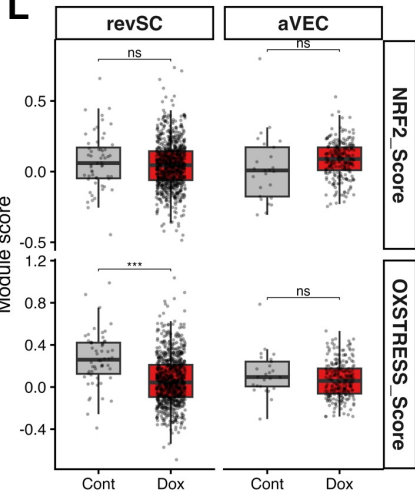

M

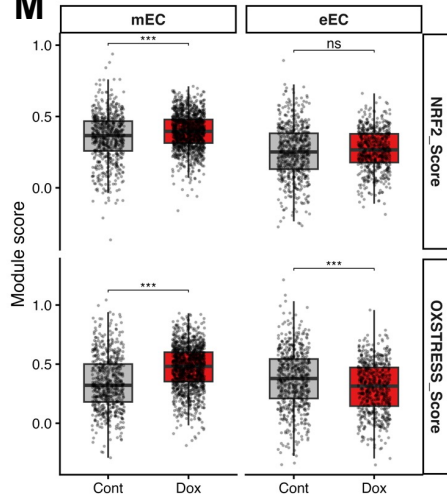

Figure S2

A

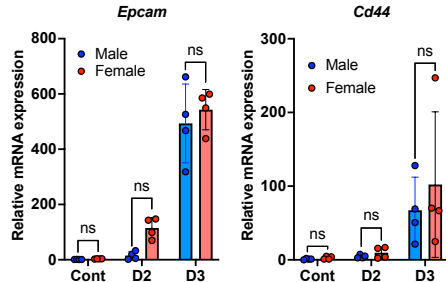

B

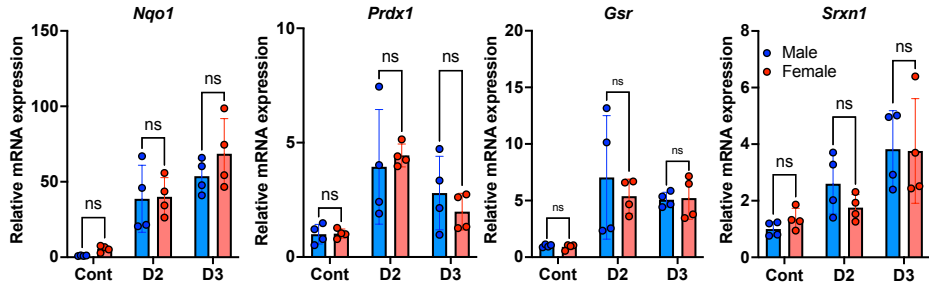

C

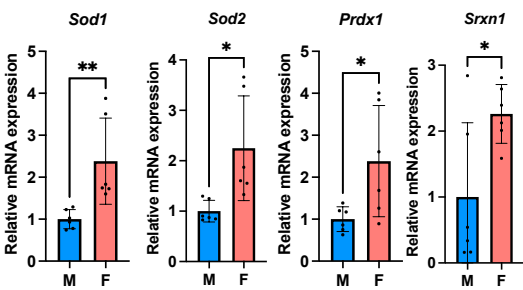

D

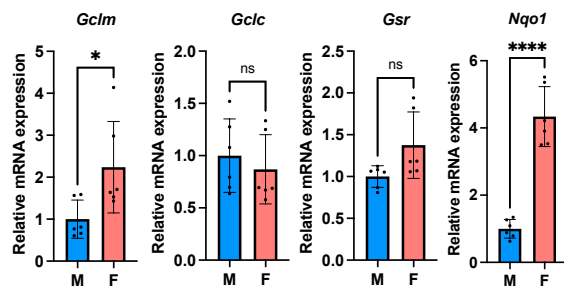

E

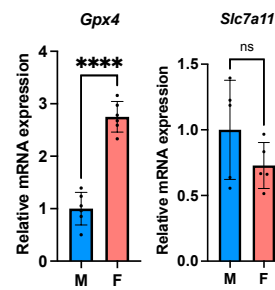

F

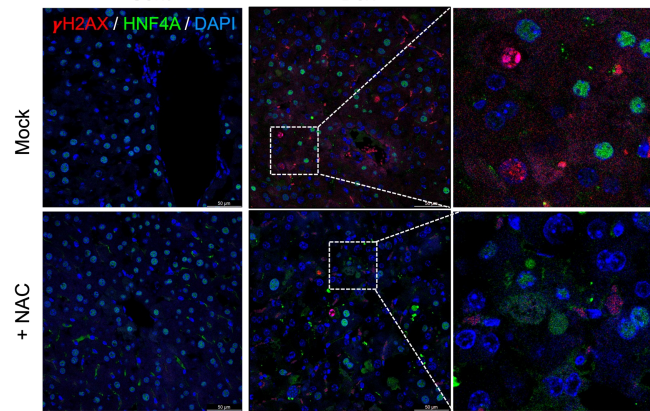

G

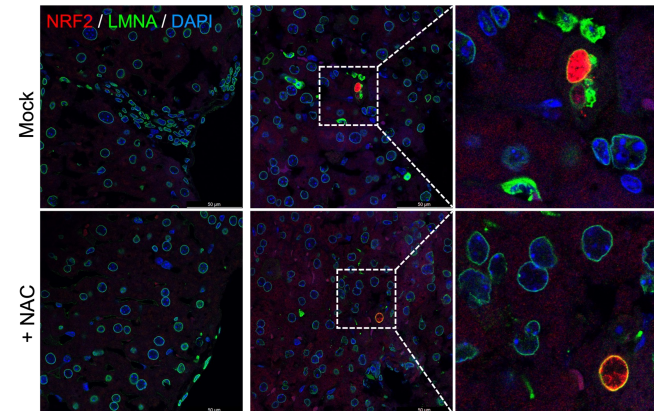

H

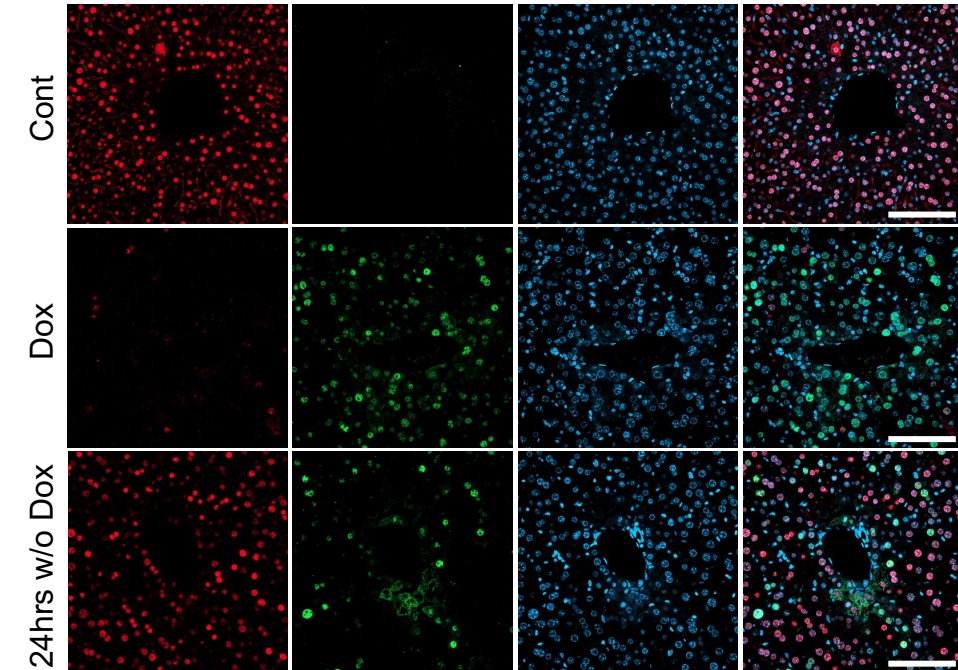

Figure S2

I

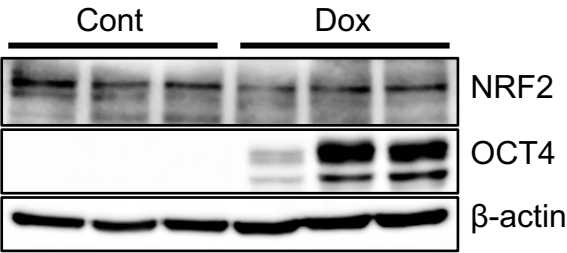

J

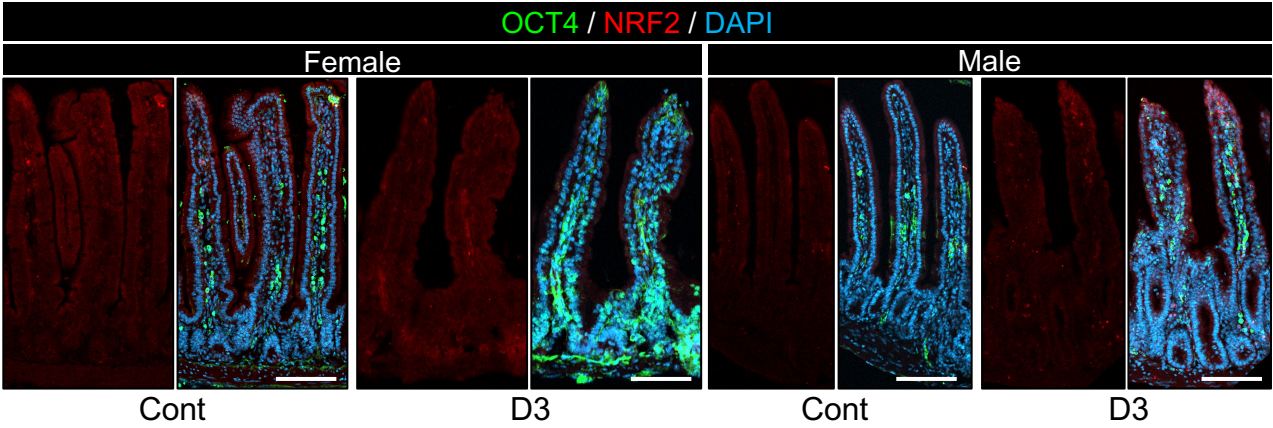

Supplement: Supplementary file 2 — Supplementary material [file mmc2.pdf]
