# Supplementary material for: Oxidative stress drives liver failure during in vivo partial reprogramming
Source: Mol Cells. 2026 Jun 4;49(8):100378. doi: 10.1016/j.mocell.2026.100378 (PMC13330666; doi:10.1016/j.mocell.2026.100378)
Supplement: Supplementary file 3 — Supplementary material [file mmc3.pdf]

Rebuttal Figure 1

(A)

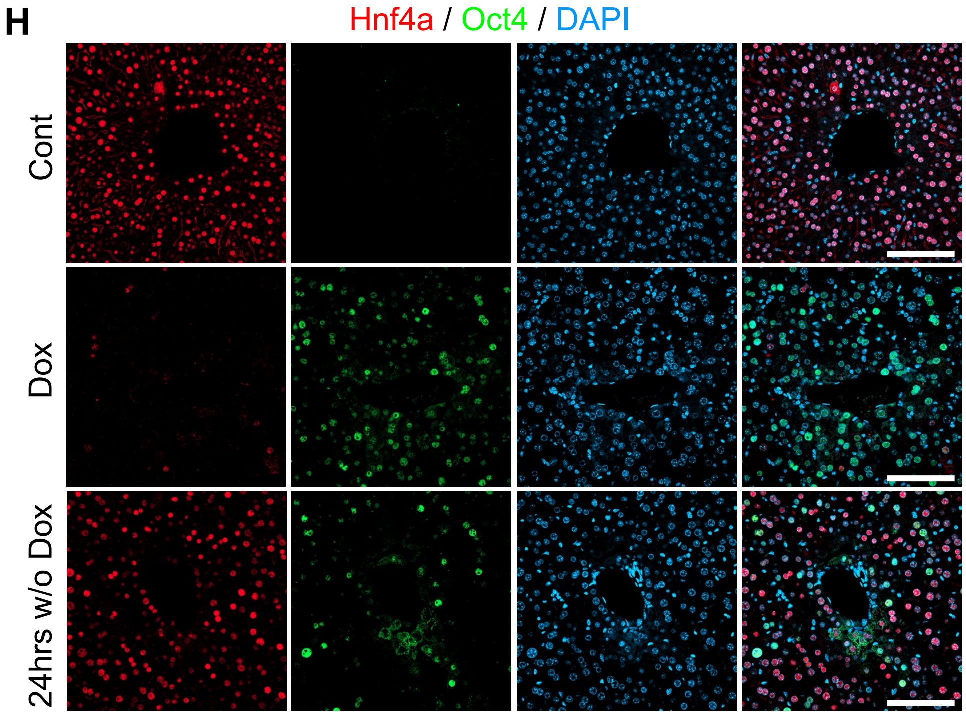

(Supplementary Fig. 2H)

Rebuttal Figure 2

(A)

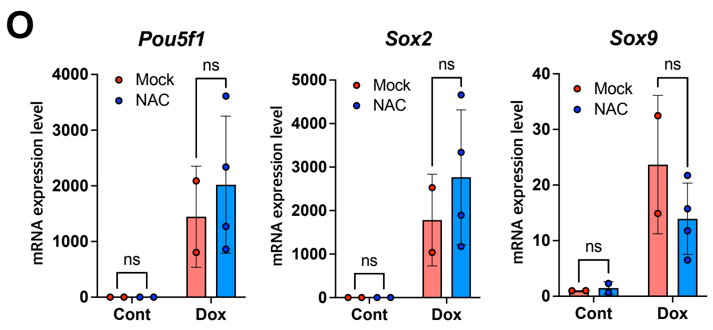

(Fig. 2O)

(B)

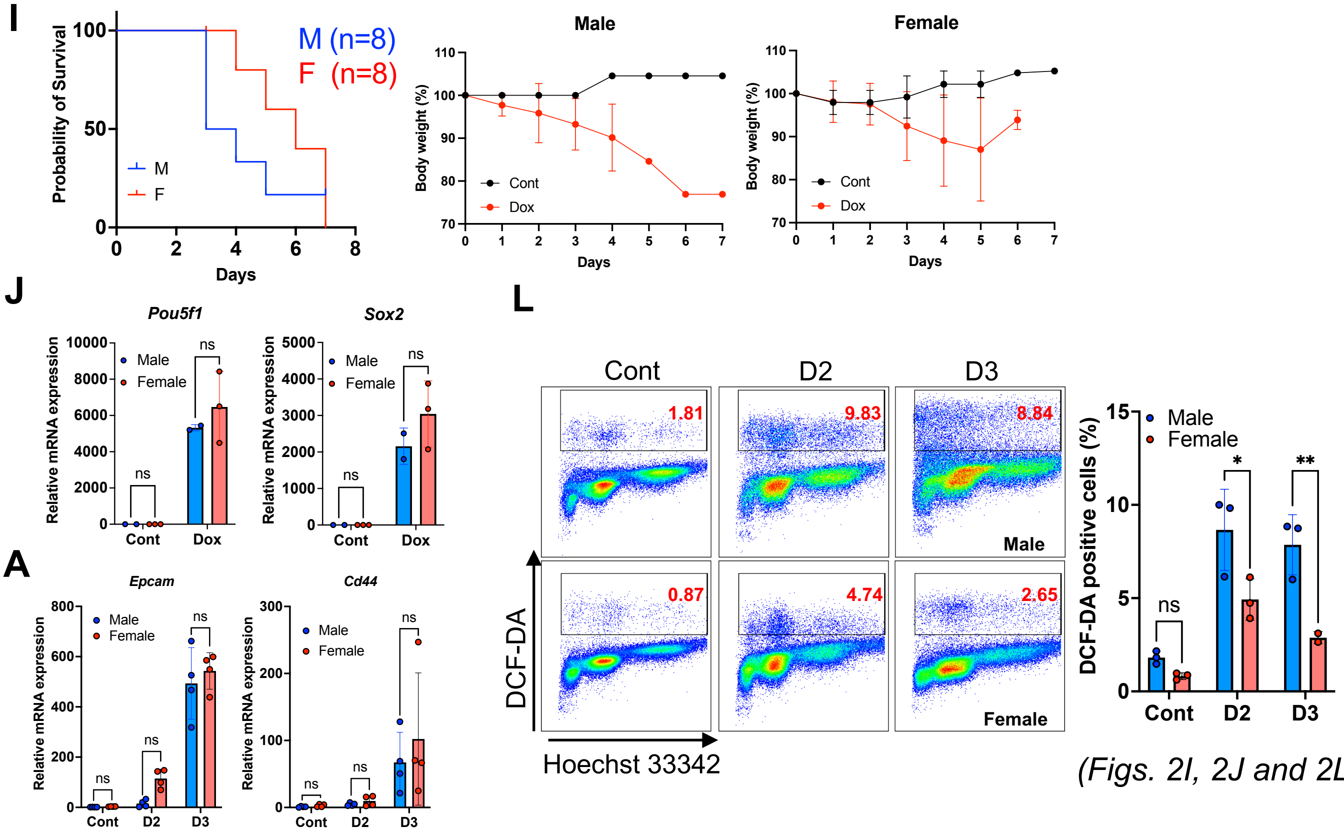

(Figs. 2I, 2J and 2L)

(Supplementary Fig. 2A)

(C)

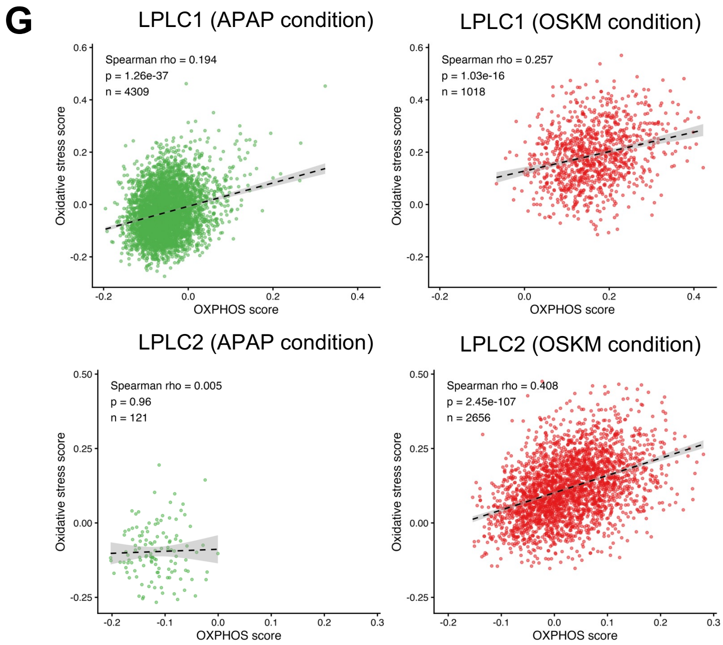

(Supplementary Fig. 1G)

Rebuttal Figure 3

(A)

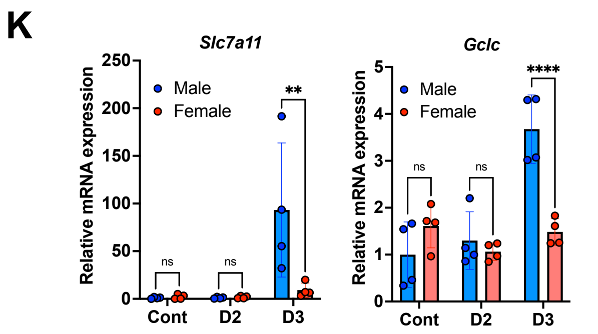

(Fig. 2K)

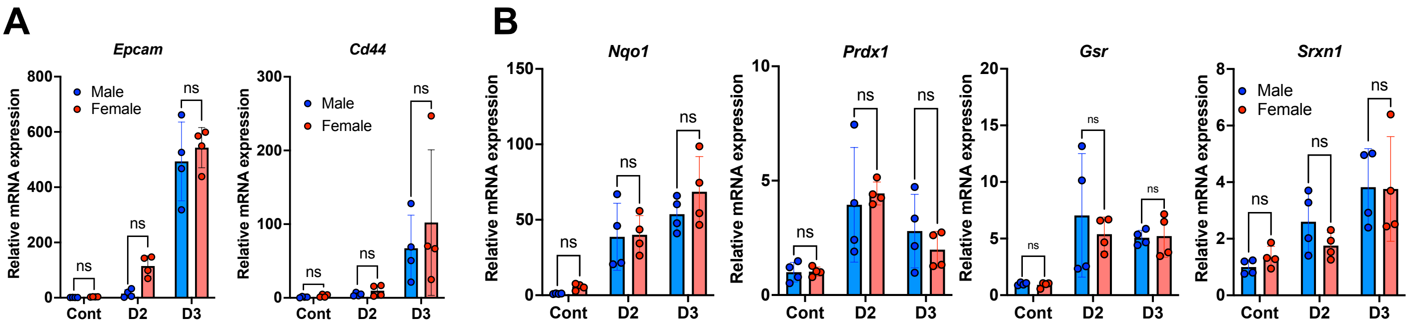

(Supplementary Fig. 2A and 2B)

Rebuttal Figure 4

(A)

F

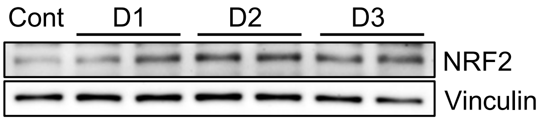

P

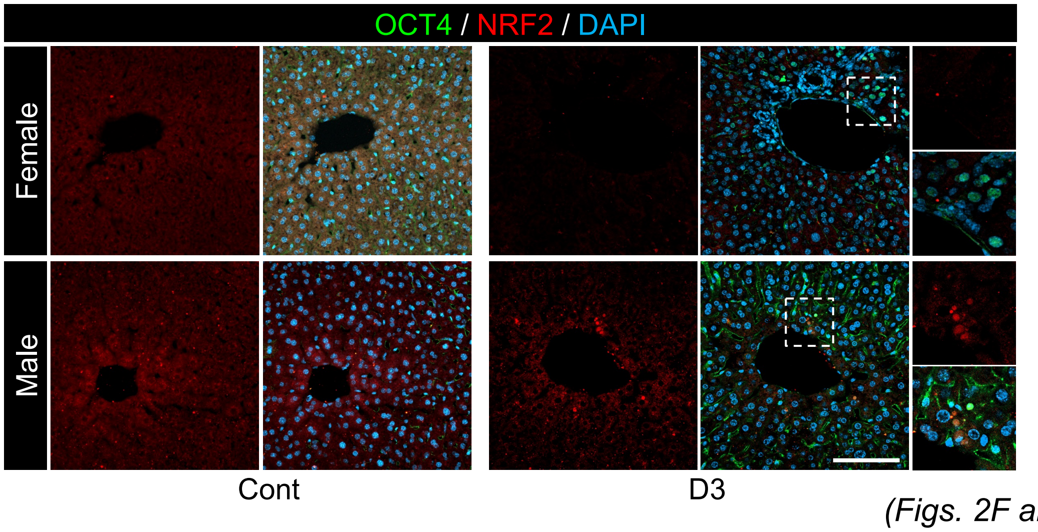

(Figs. 2F and 2P)

(B)

I

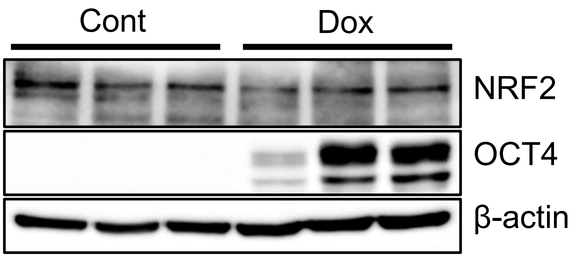

J

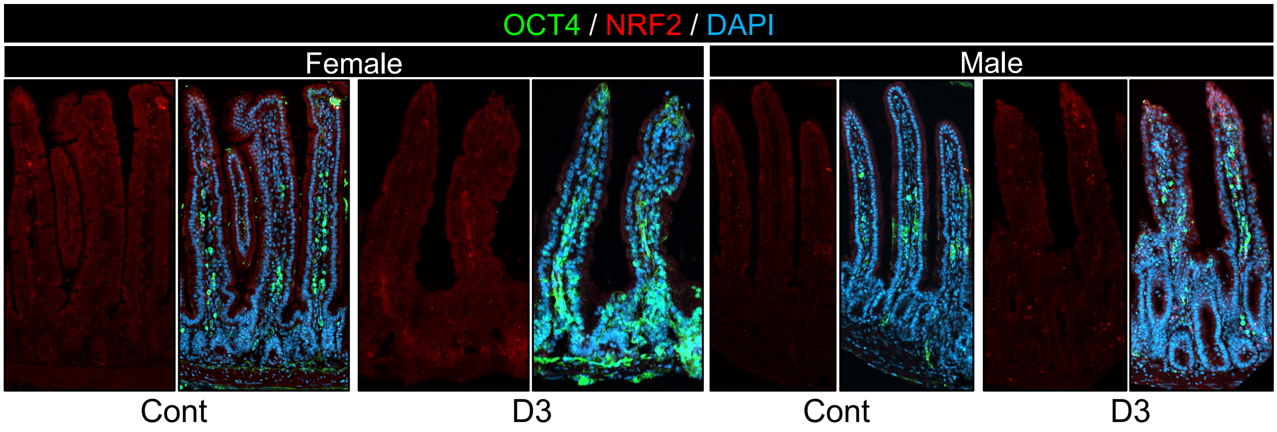

(Supplementary Figs. 2I and 2J)
